# The role of public participation in disaster risk reduction initiatives: The case of Katlehong township

**DOI:** 10.4102/jamba.v14i1.1203

**Published:** 2022-02-28

**Authors:** Ziyanda Nkombi, Gideon J. Wentink

**Affiliations:** 1 Unit for Environmental Sciences and Management, African Centre for Disaster Studies, Faculty of Natural and Agricultural Sciences, North-West University, Potchefstroom, South Africa

**Keywords:** disaster, disaster risk management, disaster risk reduction, hazard, Katlehong, public participation, risk

## Abstract

Disaster risk reduction (DRR) has become a policy priority worldwide and in line with this trend, the *South African Disaster Management Act* and National Disaster Management Framework prioritise DRR in efforts to build resilient communities with local municipalities being required to develop their own Disaster Management Frameworks. The problem is that public participation is treated as of secondary importance yet international agreements such as the Sendai Framework for Disaster Risk Reduction (SFDRR) actively promote public participation in DRR. A bottom-up approach is the most effective in ensuring successful DRR initiatives at the local level because communities take ownership of these initiatives and gain a better understanding of their risks. Community-based disaster risk reduction originated in the paradigm shift away from the traditional disaster management approach, moving away from reactive responses in the top-down approach in disaster risk management to more proactive responses. This research study explored approaches used for public participation to ensure successful DRR initiatives in Katlehong township. The study is exploratory and descriptive, having used qualitative and quantitative research approaches, which included questionnaires and interviews. The results gleaned from the data suggested that the role of public participation in DRR initiatives is ineffective in Katlehong township because of the reluctance of stakeholders to participate in DRR. Accordingly, it was recommended that the municipality host stakeholder sessions where stakeholders are informed about the role of the centre and about their own role in DRR. Such stakeholder sessions should assist in resolving issues such as confusion about the stakeholders’ roles in DRR and help to obtain buy-in from all the stakeholders.

## Introduction

Disasters are considered to be a local phenomenon because ‘local communities are on the frontlines of both the immediate impact of a disaster and the initial emergency response to a disaster’ (ed. Shaw 2012:4; UN/ISDR 2007a:iii). Increased interaction between disasters and communities have emphasised the importance of local institutions encouraging and supporting vulnerable communities to build their coping capacity despite the fact that the community must be at the centre of all solutions that are provided. The adoption of frameworks such as the Yokohama Strategy and Plan of Action for a Safer World, Hyogo Framework for Action and Sendai Framework for Disaster Risk Reduction (SFDRR) have highlighted the importance of empowering communities to reduce their own disaster risks. Community-based disaster risk reduction (CBDRR) provides a solution to the increased disaster risks within communities, as it aims to strengthen and enable communities to ‘undertake any programmes of development including disaster preparedness and mitigation’ (ed. Shaw 2012:5). However, it is important that communities have their own resources and social bonds in addition to their efforts to take part in DRR activities, because a lack of resources and social bonds may hinder their participatory efficiency (Allen 2006:84).

## Theoretical orientation of community-based disaster risk reduction

**FIGURE 1:**
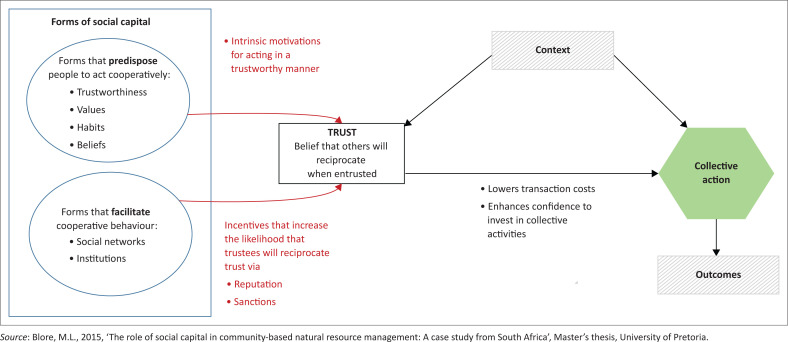
A conceptual model showing how forms of social capital generate collective action.

The social capital theory, which is ‘about the value of social networks, bonding similar people and bridging between diverse people, with norms of reciprocity’ (Claridge 2004) is considered to be an appropriate theory that underpins CBDRR because they are both concerned with encouraging the involvement of local people in identifying and solving issues in their communities (see Figure 1). The social capital theory enables individuals and/or groups through collective action to reach desirable outcomes (Silici n.d.:2). The theory also promotes a sense of belonging, valuing diversity in others and similar life opportunities (Babbie 2007:11).

The community-based approach in general emphasises the importance of the community, both as key actors in and primary beneficiaries of an initiative (Lassa et al. 2018:1–2). Thus, it is important when working with a community-related topic to constantly ask ‘what is the community?’ in question (Petal et al. 2008:193). Although communities are considered to be heterogeneous because of gender, age, experience, culture, leadership styles and religion, it is important that every individual is treated equally. It is, therefore, important to constantly identify factors such as geography, culture, community representatives (Do they exist? Have they been elected? Appointed or hereditary?), who is assumed to comprise the community (by themselves and others), and who is considered to be marginalised from the community. Although there may not be definitive answers, these questions do assist in understanding the different views and characteristics of communities, communities within communities and sectors of communities (Girvan & Newman 2002:7821).

Community-based disaster risk reduction may empower marginalised individuals (Chambers 2012) although a local focus often fails to incorporate influences from higher levels (Scoones 2009). Consequently, strategies are influenced by community members’ narrow experience of local drivers and their immediate needs and are constrained by their limited power (Conway & Mustelin 2014).

According to Abarquez and Murshed (2004), CBDRR originated in the paradigm shift away from the traditional disaster management approach. This shift prompted the emphasis to be moved away from the structural approaches to the more non-structural approaches, thus implying a move away from the reactive responses in the top-down approach in disaster risk management to more proactive responses (Scolobig et al. 2015:202). Shaw (ed. 2012:4) suggested that community-based disaster-related activities existed more than 100 years ago where communities would take care of each other to reduce damages or harm caused by disasters. After the failure of government-based DRR initiatives designed to address the needs of people and communities, CBDRR initiatives started to receive recognition at both the national and local levels of governments (Phiri 2014:29). The evolution of CBDRR began with community-based disaster management (CBDM), which gradually evolved into CBDRM and then into CBDRR (ed. Shaw 2012:4).

Community-based disaster management became popular during the 1980s and 1990s as a result of the failure of the traditional disaster management approach to address the needs and priorities of communities and to reduce their vulnerability to disasters. The latter had been primarily because of the use of a top-down approach (Phiri 2014:27).

**FIGURE 2:**
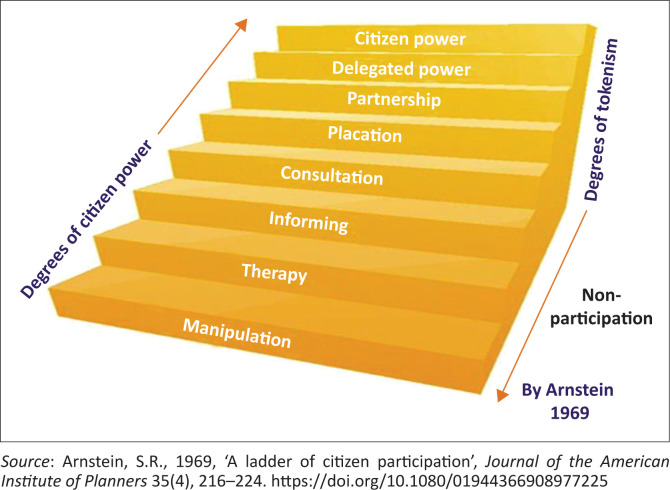
Ladder of citizen participation.

Community-based disaster risk reduction is a process of DRR that places significant emphasis on community participation, primarily because communities themselves are directly affected by disasters and the CBDRR also requires the collaboration of institutions and organisations in sectors such as health, agriculture, education and infrastructure development (Isidiho & Sabran 2016:270). This process was adopted because of the failure of disaster risk management to place communities at the centre of decision-making. In the disaster risk management approach decisions were made by government officials based on their own perception of the needs of communities (Scolobig et al. 2015:203). This would be referred to as the manipulation and therapy level in public participation in Sherry Arnstein’s (1969) Ladder of Citizen Participation (see Figure 2).

The move from CBDRM to CBDRR resulted from the increase in the loss of life and the social and economic disruptions because of disasters (Shaamhula 2015:14). The CBDRR provides a proactive approach to activities aimed at reducing risks to communities. Thus, the use of a CBDRR process became more popular as it resulted in DRR being more effective because of the use of knowledge emanating from those directly affected by hazards (ed. Shaw 2012:5). Disaster risk reduction, for this article, is defined as:

[*T*]he conceptual framework of elements considered with the possibilities to minimise vulnerabilities and disaster risks throughout a society, to avoid (prevention) or to limit (mitigation and preparedness) the adverse impacts of hazards, within the broad context of sustainable development. (UN/ISDR 2005:14)

This means that DRR has to do with the resources that stakeholders in communities have at their disposal to reduce their susceptibility and vulnerability to hazards. Accordingly, hazard, vulnerability and capacity assessments are used as strategies for DRR because they help to identify the ability of a community to reduce its own disaster risk (Mercer 2010:249). Local communities are at the forefront of both the immediate impact of disasters and the initial emergency response, which is crucial in saving lives (UN/ISDR 2007a:iii). In addition, the community-based approach provides the most trustworthy primary data in understanding a community’s disaster risk profile and offers certain benefits that enable communities to be resilient.

Benefits of the community-based approach are particularly apparent in those initiatives that aim to build resilience to both hazards and climate change as local communities are able to work with local municipal officials and identify the risks themselves, thereby addressing vulnerability issues using local knowledge and skills (Mercer et al. 2009). Projects such as the school competition on DRR knowledge, in South Africa’s Chris Hani District Municipality, which was organised to celebrate the International Day for Disaster Reduction (IDDR) in 2006, were used as a platform to launch disaster reduction initiatives in the country (UN/ISDR 2007b:30–32). The initiative comprised a school competition using art, music and drama to portray the impact of disasters on communities, how these disasters could be prevented and the role of communities in increasing their own resilience to disasters. These benefits include the provision of information and education to vulnerable communities to act in reducing the risks they face, thus enabling communities to take control of their own fate. Communities acquire knowledge about disaster risk management, which contributes to their understanding of their own disaster risk profile and, thus, enable them to utilise their coping capacity and skills using the knowledge they acquire from the other stakeholders in order to find solutions to their challenges.

## Contextualising the role of public participation in disaster risk reduction

Existing literature on social science, communication and political science has highlighted various terms such as ‘community’ and ‘public and stakeholder participation’, among others, as relevant in the engagement between decision-makers and citizens (Burnside-Lawry & Carvalho 2015:83) while a sociological study by Taylor (1994:117) referred to community participation as a process, which is informed by the objectives of the community. These objectives include empowering the community members to be able to act on their own to ensure effective community development. According to Bayat and Meyer (1994:156), community participation is the ‘act of taking part or the involvement of community members in specific community activities’. Jacobson and Servaes (eds. 1999:1) defined the term from a development communication perspective and referred to public participation as the notion of involving the public in planning for the improvement of their living conditions, as well as the notion of placing the public at the centre when promises of betterment are being conceived. According to the EPA (2017):

[*P*]ublic participation is a process, not a single event, consisting of a series of activities and actions by a sponsor agency over the full lifespan of a project to both inform the public and obtain input from them. (n.p.)

Thus, this process empowers stakeholders, such as individuals, interest groups and communities and provides them with an opportunity to influence the decisions that may affect their lives (EPA 2017). Mosotho (2013:11) added that public participation is a process whereby ward councillors and ward committee members attempt to involve entrepreneurs, traditional leaders and community members, among others, in the development planning process taking place in local municipalities. Stakeholder participation involves the collaboration of various actors who share the same interest that may be achieved and realised only when such actors work collectively. It is imperative that this arrangement is trust based to ensure that individual and group characteristics, which influence or are influenced by organisational behaviours and actions are recognised, analysed and examined (Mainardes, Alves & Raposo 2012). From the given definitions, public participation is considered to be a process that an institution or organisation undertakes to consult with interested or affected individuals, organisations, business and/or government entities before making a decision that either directly or indirectly concerns the public. For this study the term ‘public participation’ was used because ‘community participation’ refers to the participation of members of a particular community (Masango 2001:106). In public participation, the word ‘public’ is not ‘identified on the basis of specific and fixed characteristics’ (Masango 2001:107) because public participation involves all members of the public who are interested in issues that are at stake (Masango 2001:108), for example, the issue of reducing risks in vulnerable communities. In addition, public participation helps to prevent or minimise disputes that may arise regarding the issue at hand. Public participation has become popular in environmental science as, owing to the nature of hazards, environmental science experts have recognised the need to embrace the elements of deliberative and pluralistic participation models in order to mobilise the interests of individuals in discussions on disaster management (Habermas cited by Rood 2012:49).

Sherry Arnstein’s *Ladder of Citizen Participation* published in 1969 depicts the degrees of citizen involvement (Arnstein 1969:217). Figure 2 illustrates that, at the ‘manipulation’ and ‘therapy’ level, public participation is merely pretence and the public is simply informed about what has been decided or has happened. The ‘informing’, ‘consultation’ and ‘placation’ levels are where the public is provided with information about a project or issue and is asked to comment and give advice. However, the public’s input is not considered when the final decision is taken and they may not even be given feedback as to the reasons why a particular decision was taken. The ‘partnership’, ‘delegated power’ and ‘citizen power’ levels depict how the participation of the public moves from their inputs influencing decisions taken by government officials to being given delegated power to take decisions. The ladder of citizen participation then moves to where the public participates by taking initiatives independently of external institutions in relation to the resources and technical advice, they require but the public retains control over the way in which the resources are used.

According to Chen, Lui and Chan (2006:210), public participation in DRR measures goes as far back as World War II when the public provided emergency services as a result of the lack of professionals during that period. In addition, between 1960 and 2006, Taiwan’s community-based disaster management programme involved firefighting agencies calling for individual volunteers and providing them with basic response skills to assist with emergency response (Chen et al. 2006:213). Subsequently, in 1982, the Office of the United Nations Disaster Relief Co-ordinator (OUNDRC) published the Disasters and the Disabled Manual (OUNDRC 1982:1). The manual encourages the engagement of families and communities in responding to the needs of people following a disaster (OUNDRC 1982:34). In 1993, the IDNDR Aichi/Nagoya International Conference urged that the response of local administrations to natural hazards should be supported by community members, corporate institutions and non-government organisations (NGOs) (IDNDR 1993:7).

However, such support became more formalised in 1994 through the Yokahama Strategy and Plan of Action for a Safer World, in which the community’s involvement and participation was encouraged in order to gain greater insight into individual and collective perceptions of development and risk. It also encouraged disaster management officials to gain a clear understanding of the cultural and organisational characteristics of each society, as well as its behaviour and interactions with the physical and natural environment (IDNDR 1994). The Hyogo Framework for Action (UN/ISDR 2005) also placed emphasis on the involvement of communities by stating that:

[*B*]oth communities and local authorities should be empowered to manage and reduce disaster risk by having access to the necessary information, resources and authority to implement actions for disaster risk reduction. (p. 5)

The development of public participation in DRR culminated in 2015 at the Third United Nations World Conference on Disaster Risk Reduction (WCDRR), where the SFDRR 2015–2030 was adopted (UN/ISDR 2016). The SFDRR focuses on four priorities aimed at reducing disaster risks worldwide. Its fourth priority is aimed at ‘enhancing disaster preparedness for effective response and to “Build Back Better” in recovery, rehabilitation and reconstruction’ (UN/ISDR 2016). In order to achieve this priority, nations are called upon to promote the cooperation of various stakeholders in communities in post-disaster reconstruction initiatives (UN/ISDR 2016). Disaster risk reduction requires multisectoral and multistakeholder actions in order to manage disaster and climate risk, which supposes a strong governance system characterised by relevant laws and policies, institutions and coordination mechanisms, strong leadership, clear roles and responsibility, resources, monitoring and accountability set up across all sectors, all actors and all levels (UNDRR 2019:16).

Public participation in DRR ensures shared responsibility and transparency in DRR planning and implementation. It also allows decision-makers to make communities aware of the risks with which they are faced while informing the community about their own understanding of such risks. This then enables the creation of an environment in which both parties reach a consensus on reducing the risks (Forbes-Biggs 2011:6; Reddy 2010:44–45). However, communities in high-risk areas are often largely excluded from formal governance processes and services despite the fact that community-led planning is essential for identifying and monitoring hazards, reducing risks and preparing for disasters (Parnell, Simon & Vogel 2007:358).

## Research setting

**FIGURE 3:**
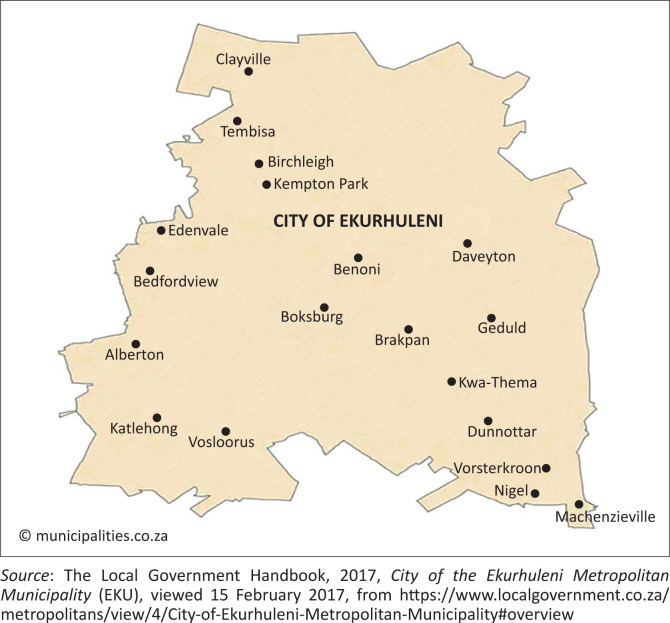
Map of the City of Ekurhuleni Metropolitan Municipality.

Katlehong township is situated in the Ekurhuleni Metropolitan Municipality (EMM) (see Figure 3) in Gauteng and was established in 1945 with a population of 407 294 and 124 841 households (StatsSA 2011). It is characterised predominantly by informal settlements, overpopulation, poverty and unemployment (The Local Government Handbook 2017). It is also considered to be one of the most poorly served townships when it comes to basic services such as water and sanitation and electricity in the EMM, as well as in Gauteng as a whole (The Local Government Handbook 2017). Mining-related risks such as sinkholes, flooding and fires are also predominant in the area (Myburgh & Bodenstein 2015:18). All these factors indicate the area’s vulnerability to hazards and lack of capacity to ensure local community resilience (Strydom 2008:38). Moreover, it is important to ensure that the residents of the township are involved in the planning, development and implementation of the DRR initiatives undertaken in the area. Consequently, it will assist in building a community that is resilient to hazards and also help to inform the public of the underlying scientific facts through education provided by disaster management officials. In addition, disaster management officials will be aware of the coping mechanisms used by community members to reduce the impact of disaster on the area (Kasemir et al. 2003:7). However, the data available at the time of the study showed that, in this area, the members of the public are not given a platform to voice their understanding of disaster risks in their area (Strydom 2008:50).

## Methodology and methods

This study was both exploratory and descriptive in nature. An exploratory research design is aimed at exploring a topic and gaining an insight into a particular situation or phenomenon, while a descriptive research design is aimed at describing and providing specific details about a situation or event (De Vos et al. 2011:95–96).

Qualitative and quantitative research approaches were used to obtain a holistic understanding of public participation and DRR. The qualitative research design is used in the ‘study of a social phenomenon that is usually rooted in a literature review, which attempts to gain a holistic understanding of the phenomenon’ (Mouton, Auriacombe & Lutabingwa 2006:580) while the quantitative research design is applicable to studies in which ‘the findings are expressed in statistical data, which has numerical value such a design may comprise experimental and non-experimental designs’ (De Vos et al. 2011:145–157). This study used both approaches to assess prevailing opinions and beliefs in relation to the role of public participation in DRR initiatives. In order to do this, questionnaires and semi-structured interviews were used.

Cluster sampling is used when a sampling frame, such as a list of names, is not available and only a map of the relevant geographical area is available. This method has the advantage of concentrating the field of study in a specific section of the greater geographical area, thus saving costs and time. The researcher should, therefore, attempt to retain the clusters of areas, which are naturally grouped together, such as suburbs or street blocks. Each cluster on its own must represent the whole population and variations between the clusters must be small (McBurney & White 2004:230). For this study, the research area was clustered according to customer care areas (CCA), namely Katlehong 1 CCA and Katlehong 2 CCA. In each section, 21 respondents were identified, which included informal and formal business owners, residents in formal and informal settlements and non-profit organisations.

Snowball sampling was used for the semi-structured interviews. According to this method, an interconnected group of people refer the researcher to other members of the same group to enable the researcher to acquire more information than may otherwise have been the case (ed. Maree 2010:177). This means that snowball sampling is where research participants recruit other participants for a test or study. It is used where potential participants are hard to find. For this study, the EMM Disaster Management Unit Manager was contacted who then referred the researchers to other possible respondents with the same characteristics as the unit manager such as the officials from the Department of Energy, Community Safety, and Health and Social Development.

## Self-administered/individually administered questionnaires results and discussion

**FIGURE 4:**
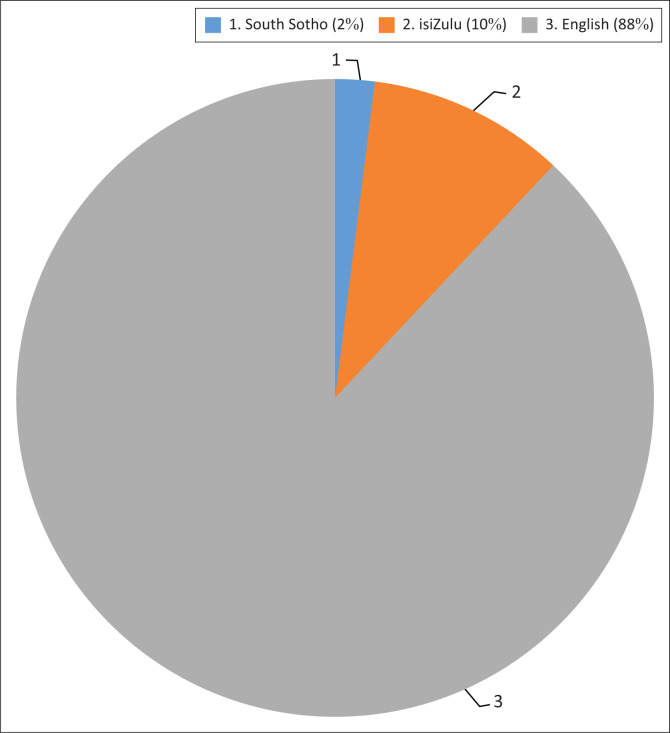
Languages used when administering questionnaires.

Questionnaires are regarded as the most popular instrument used for data collection (McBurney & White 2004:238). The questionnaires employed in the study were in English, South Sotho and isiZulu in order to accommodate the most used languages in the area. The questionnaires were completed by 42 respondents – 37 answered in English, four answered in isiZulu and one in South Sotho (see Figure 4). The results obtained from the questionnaires are discussed in the following sections.

### Community understanding of and perspectives *on public participation and disaster risk reduction*

The views of all the respondents corresponded to the definitions of public participation provided here and made reference to one or more elements of public participation, as discussed in the literature review. These elements included engaging the community, the development of the community and the community taking part in the decision-making processes. One respondent even mentioned one of the benefits of encouraging public participation as being the fact that the people take ownership of their community and its development.

**FIGURE 5:**
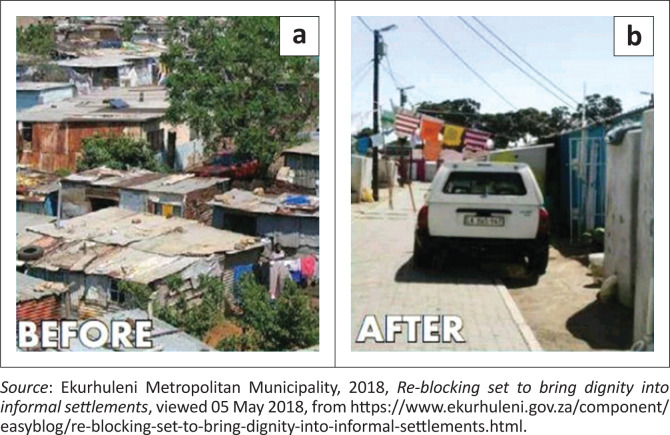
Photograph showing informal settlements before (a) and after (b) re-blocking.

The majority of the respondents’ views on DRR concurred with the definitions provided in the literature review. Some respondents even provided examples of activities that were being undertaken and/or which should be undertaken. For example, two of the participants mentioned the re-blocking system (see Figure 5) that has been used by the EMM since 2016 to limit the rapid spread of fires over large areas in informal settlements. One respondent also referred to the issue of power failure (usually caused by the explosion of transformers resulting from illegal connections) that often occurs during the winter season and could be attended to before the onset of winter. In addition, power failure leads to the use of dangerous forms of heating such as the open fires and coal fire drums (*imbhawulas*), which are often used in township homes and informal settlements and are a fire hazard.

The views of some respondents from the informal settlements did not correspond to the definition of DRR provided in the literature review. For example, one response was that DRR takes place when ‘the community is denied help by the government’. Other responses from the respondents in the formal sections of the township included that DRR means that ‘they are safe and protected’, ‘it is when you put yourself on the safe side’ and ‘it is how the community may control the disaster risk’. However, the majority of the respondents were able to provide answers that correlated with the given definition of DRR that illustrated ways in which disaster risks were being reduced in their community.

The majority of the respondents’ views on risk and hazard did not correspond to that which is provided in the literature review. Their responses included the following: ‘A risk is when you stay in a community that does not know its issues’, ‘a risk is doing something you have never performed before while knowing it might backfire’ and ‘a risk is going beyond your normal capabilities and ability’. The respondents referred to a hazard as an event that occurs when the parents and the community are not together, that is, when you do something without thinking about it or that is to be protected by public organisations or government. A few of the respondents’ views corresponded to the definitions of a risk and hazard provided here while some were only able to provide examples of a risk and a hazard. For example, they knew that a risk may constitute illnesses caused by water and air pollution while a hazard may involve hazardous chemicals and civil unrest. However, 12% of the respondents who answered the questionnaires in South Sotho (2%) and isiZulu (10%) repeated their answers regarding the two terms because a risk and a hazard are referred to by the same term in both South Sotho and isiZulu (see Figure 4).

All of the respondents’ views matched to a degree with the definition of a disaster provided in the literature. One respondent referred to a disaster as an act of God or providence and went on to say that there are also man-made disasters. This is interesting because the latter notion is scientific while the former is based on beliefs and religion. Other responses included that a disaster is an event that brings challenges to the community, such as a lack of housing resulting from damage to the houses; a disaster is an event such as thunderstorm and when there are floods; it is something that happens unexpectedly, such as a fire, which may cause harm and it is something that is beyond our control. In addition, some of the respondents were able to provide examples of hazards such as earthquakes, a stampede at a stadium and violent service delivery protests.

The majority of the respondents were aware that the EMM conducts awareness campaigns regarding risks within the community. They also indicated that they found these awareness campaigns useful because it educates members of the community who do not possess the necessary knowledge, thus helping the community to stay safe. One of the respondents also mentioned that the awareness campaigns are not only conducted by the municipality but also by other stakeholders. The respondent added that ‘some of the community members do not listen because they want tangible things such as houses and roads’.

From the given discussion one can see that the community of Katlehong has a good understanding on public participation and DRR, which is a result of the municipality’s awareness campaigns. However, it is not enough for them to only have an understanding and perspectives of public participation and DRR, they also need to take up their role.

## Role of public participation in disaster risk reduction initiatives

**FIGURE 6:**
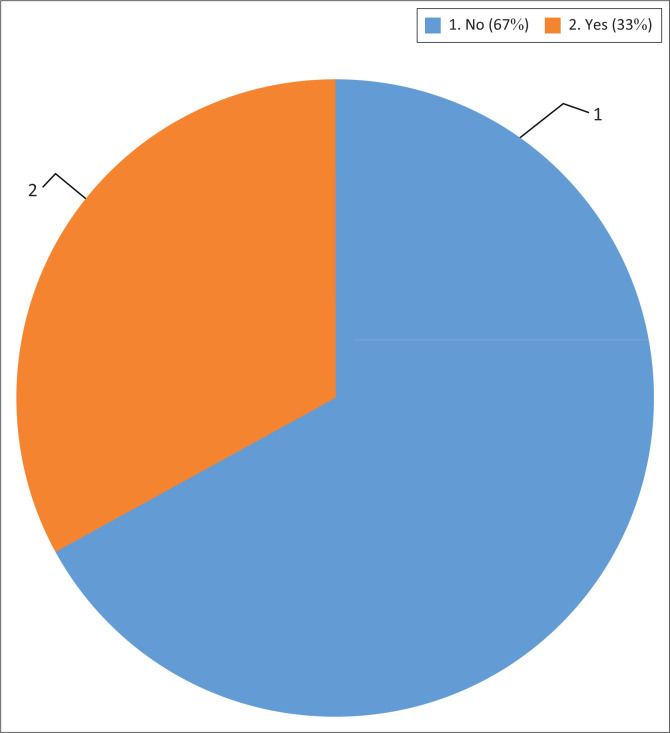
Community knowledge on public participation.

As Figure 6 indicates, 67% of the respondents were not aware that they were permitted to participate in the designing, planning and implementation of DRR initiatives in the municipality while, on the other hand, 33% of the respondents were aware that they were permitted to participate in these initiatives. The latter group had found out about this in various ways, which included a stakeholder meeting, at schools and from the Ekurhuleni Metropolitan Police Department (Social Crime Unit) although one of the respondents mentioned not recalling how this was found out.

**FIGURE 7:**
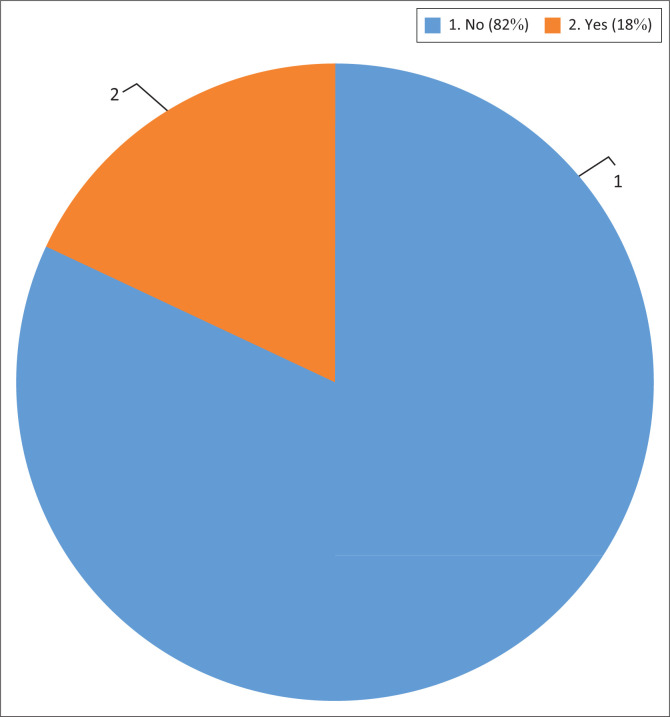
Community knowledge on legislative frameworks for public participation.

A total of 82% of the respondents were not aware of the legislative frameworks that enable public participation (see Figure 7) while 18% of the respondents were aware of these frameworks. Such frameworks include the Constitution and the local government: Municipal systems framework.

**FIGURE 8:**
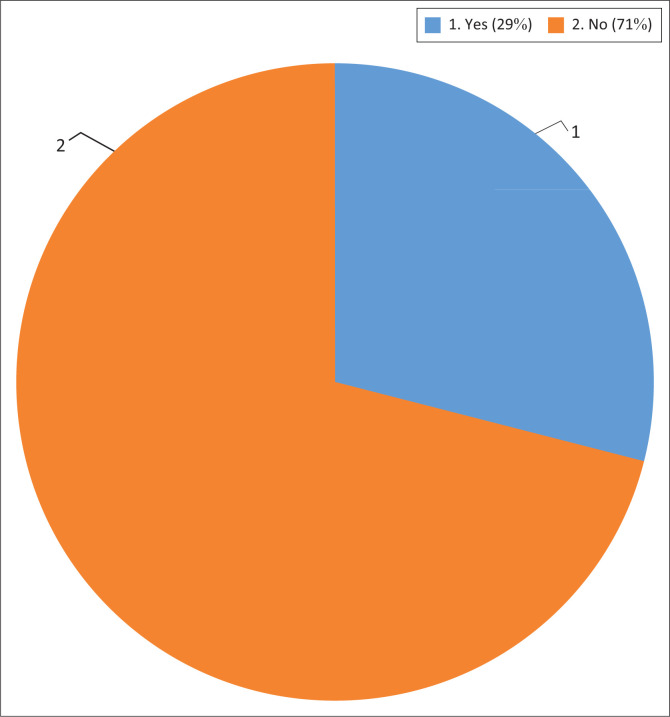
Community participation in disaster risk reduction activities.

Nearly 71% of the respondents had never participated in the designing, planning and implementation of DRR activities (see Figure 8). They indicated the reason for this was that they were not aware that they could participate or how they could participate. The remaining 29% of the respondents had participated and stated that they had performed because they wanted to make an impact in their community. In addition, they also wanted to assist their local government. Through the municipality’s public participation programmes, they had seen the importance of reducing the disaster risks in their community. All of the respondents who had participated in the implementation of DRR activities had received training in public participation. These respondents were of the view that their participation had been valuable because it had reduced the risks that they faced in their community. They felt they had added value to the government’s objective of reducing risks in their community by sharing their knowledge and their skills during workshops held in their CCA. One of the respondents (a ward councillor) indicated that participation by ward councillors encourages other community members to participate because they realise that a particular project is significant for them.

It is evident, from the given discussion that a lot more needs to be performed when it comes to engaging the public in the designing, planning and implementation of DRR activities. Although this might be difficult because ‘recovery literature does not often make a distinction between public participation in recovery activities and public participation in decision-making for recovery’ (Vallance 2015:1298).

## Semi-structured interview results and discussion

The following sections present the results from the semi-structured interviews, which were conducted with five municipal officials and political office-bearers from the EMM.

### Understanding of and perspectives on public participation and disaster risk reduction

The understanding and perspectives of these respondents regarding public participation were overwhelmingly unanimous. The results obtained from the completed questionnaires discussed in the given section were supported by a statement from one of the respondents who mentioned that ‘public participation refers to the local government taking into consideration the needs of the communities and also taking into consideration the knowledge community members have’. This view is aligned with the fact that local government resources are aimed at improving the lives of the people in communities, thus when the community members are included this means they are in a partnership with local government. Furthermore, public participation involves the municipal officials consulting the community, thereby indicating that the officials value the input provided by the community. When community members are given the opportunity to guide municipal officials in relation to how they are able to cope with disaster risks, municipal officials should incorporate the community members’ skills and knowledge into the DRR initiatives.

However, the study found that the participants’ understanding and perspectives on DRR varied although they did concur that DRM should be given more attention, especially because it would seem the focus tends to be more on pre-disaster, especially when differentiating between a risk and hazard. The respondents from the municipal departments were of the view that a risk and a hazard are the same thing. One of the respondents, a disaster management specialist, indicated that a risk is illustrated by the equation thus implying that a risk refers to event/incident, which has the potential to cause harm, danger or loss. This meaning of risk is similar to that of a hazard because, when using the progression of vulnerability (Wisner et al. 2004), a hazard also has the potential to cause harm when it interacts with other factors such as root causes and dynamic pressures.

All the respondents agreed that the EMM: Disaster Risk Management Unit conducts community awareness campaigns in Katlehong township although some of the respondents, who were municipal officials, indicated that they had not been involved in these campaigns. Other respondents expressed the view that the aim of awareness campaigns is behavioural change, which cannot be measured.

One of the respondents indicated that the community members no longer trusted the government as a result of their perceptions of unfulfilled promises and, consequently, community members just no longer care about having a partnership with the government. This was the case especially when municipal officials do not have something tangible, such as housing, to give to community members. A municipal official cited an example of how the priorities of community members differ from those of municipal officials, for example, at a safety awareness campaign, the community had wanted to address the issue of roads in their region. In other words, the community members were using every chance they were given to interact with municipal officials to address the dynamic issues in their communities, regardless of the fact that the officials had been there only to create awareness. However, the officials had found another way of creating public awareness with the municipal officials conducting awareness campaigns at schools where they gave the learners squeeze bottles, backpacks, pencil cases and lunch boxes with emergency numbers on them in the hope that the children would relay the message of DRR when they went home.

One can note that from the given discussion the municipal officials and political office-bearers’ understanding and perspectives was unanimous. They all were aware that the EMM: Disaster Risk Management Unit conducts community awareness campaigns in Katlehong township.

### Legal frameworks enabling public participation and disaster risk reduction

This research study revealed that the majority of the respondents were aware that the municipality had a disaster management plan but did not know that each of the municipal departments was also required to prepare a Disaster Management Plan. Section 52(1)(a) of the *Disaster Management Act* (57/2002) (DMA) provides that ‘each municipal entity … must prepare a disaster management plan’ (South Africa 2002:52). The National Disaster Management Framework (NDMF) provides for a phased approach to disaster risk management planning and implementation. The majority of the respondents were not able to answer the question: To what degree does the EMM adhere to the legal requirements provided in Section 53(1)(d) of the DMA in the preparation of its disaster management plan? (Section 53(1)(d) refers to consultation with the local community on the preparation or amendment of the disaster management plan.) During one of the interviews, a Disaster Management Specialists at a Disaster Management Centre in the EMM indicated that the municipality does have disaster management plans for all the departments in place and that every department was aware of the need to have a Disaster Management Plan, the interviews held with the other municipal officials proved this not to be the case.

According to the Green Paper on Disaster Management (Republic of South Africa 1998:17), disaster management and risk reduction policies must be transparent and inclusive in the way in which decisions are taken and also how information is exchanged and the way in which stakeholders are consulted on the implementation of such policies. Section 47 of the DMA (Republic of South Africa 2002:46–48) provides that a municipal disaster management centre must be set up to provide the necessary guidance to organs of state, the private sector, NGOs, communities and individuals in the municipal area in order to assess and prevent or reduce the risk of disasters, as well as promote both formal and informal initiatives that encourage risk-avoidance behaviour by the stakeholders in the municipal area. The given provision in the DMA was supported by the respondents as they were in consensus that the factoring in of public participation in DRR policies and the implementation thereof had to be given priority. Although one of the respondents indicated that the Disaster Management Advisory Forum sat on a quarterly basis, there was nevertheless still a need to obtain the necessary buy-in from some of the municipal departments and the community because these actors were not interested in participating while there was clearly also some resistance from municipal departments and communities to factoring disaster management into their work or institutions.

The majority of the respondents were unable to identify any gaps and/or challenges in ensuring an awareness of the importance of public participation in DRR policies because they were not familiar with the policies. This finding contradicts the emphasis the SFDRR (UNISDR 2016:13) places on the involvement of communities when it states that ‘it is necessary to empower local authorities and local communities to reduce disaster risk, including through resources, incentives and decision-making responsibilities’. In view of the fact that disaster management is a multisectoral/multidisciplinary function the perception that disaster management officials are solely responsible for disaster management was one of the issues that was pointed out by a respondent because everything related to disaster management is always referred to the Disaster Management Officials. The respondents also expressed the view that, at the time of the study, a poor relationship existed between communities and the government and identified a lack of understanding of the roles and functions of stakeholders as being among the challenges faced in this regard.

The given discussion suggests that all the municipal departments need to familiarise themselves with the legal frameworks that enable public participation and DRR in order to know their role in DRM.

### Role of public participation in disaster risk reduction initiatives

According to a disaster management specialist respondent, the EMM Disaster Management Centre had identified the top 16 hazards by the means of a Vulnerability and Risk Assessment, which had been conducted in the EMM. This list included the hazards to which communities are vulnerable per CCA with Katlehong 1 CCA being deemed to be vulnerable to hazards such as civil unrest, floods and motor vehicle accidents, while the hazards to which Katlehong 2 CCA was vulnerable included pest infestation, hazmat and civil unrest. In order to manage these hazards, they have been allocated to the relevant stakeholders or departments. A respondent employed in the Department of Roads and Storm Water indicated that the EMM Disaster Management Centre informed the department about hazards relevant to the department, such as flooding caused by blocked storm water systems. Workers would be sent to unblock those storm water systems prior to the rainy season. In addition, a respondent in the Department of Health and Social Development indicated that the Environmental Health Department, for example, focused on pest infestation and food safety, among other things. In addition, with regard to food safety, this department conducted inspections of both formal and informal businesses that dealt with food in Katlehong while the department also conducted awareness campaigns with community members in general regarding food safety, although this was still in its early stages. The aim of this initiative was to prevent and mitigate the outbreak of diseases such as listeriosis.

Respondents further indicated that community members were not interested in participating in DRR activities because they longed to see tangible benefits such as infrastructure. However, during the data collection, some of the respondents from the community indicated that they were interested in participating in DRR activities but did not know how and where they could do so. The respondents suggested that the reason why they are perceived as being ignorant about participating in community initiatives was because the people had been made promises that had not been fulfilled and had thus lost faith in the government.

Nevertheless, EMM Disaster Management Centre had established volunteering initiatives such as Humanitarian Assistance Response Teams and Community Emergency Response Teams in every township in the EMM. The team members are trained to be the first respondents when disasters occur. These Humanitarian Assistance Response Teams had been established in April/May 2018 and consisted of school children. These children were trained in the hope that what they would take home and share what they had been taught. This initiative represented an attempt to help communities to obtain a clear understanding of disaster management because there is much they can do on their own to reduce disaster risks. In addition, they would then be in a position to teach other stakeholders about what they had been doing, thus providing these other stakeholders with an opportunity to bring their own expertise to DRR. The Green Paper on Disaster Management provides that:

[*C*]ommunities must know … what their own responsibilities are, how they can help prevent disasters, how they must react during a disaster (and why) and what they can do and support themselves and relief workers, when necessary. (Republic of South Africa 1998:17)

Although from the given discussion it is indicated by the municipal officials that volunteering initiatives and awareness campaigns are established and implemented, it seems like the public is not involved in the designing, planning and implementation of DRR activities in the township. This is because the municipal officials have indicated that they were not aware if the public had become involved in the implementation of DRR activities through other stakeholders and were not interested in participating in DRR activities because they longed to see tangible benefits such as infrastructure. This challenge can be attributed to the lack of service delivery as mentioned here.

## Conclusion

Public participation is considered to be one of the cornerstones of effective DRR (Reddy 2010:43). Legal instruments such as the DMA, the *Disaster Management Amendment Act* (16/2015) and the NDMF of 2005 call for the prioritisation of DRR and the engagement of communities. At the time of the study the EMM had both a Disaster Management Plan and a Disaster Management Framework in place, both of which are promulgated by these legal instruments.

It emerged from the literature review that although public participation is considered to be one of the cornerstones of effective DRR and there are policies and/or plans in place, communities in high-risk areas are often excluded from the formal decision-making processes regardless of the fact that disaster risks have been magnified by the increase in vulnerabilities related to underdevelopment and climate variability, among others (UN/ISDR 2005:1).

The data gathered suggested that public participation in DRR initiatives in Katlehong was focused primarily on informing and consulting the public. The initiatives that have been implemented are focused on providing understanding and education to communities. Moreover, it was found that the stakeholders did not fully understand their roles or how they fitted into disaster risk management. This was identified because some of the respondents referred the researcher to the Disaster Management Centre when they heard the term DRR as they were under the impression that they did not have a role to play in DRR. It is, thus, important that all stakeholders be informed of their roles by means of a stakeholders’ session because it is imperative that all stakeholders are involved if disaster risks are to be effectively reduced.

## Recommendations

In line with the research findings, the following recommendations are suggested:

- Stakeholder sessions should be hosted by the Municipal Disaster Management Centre where stakeholders are informed about the role of the centre and about their role in DRR. Such stakeholder sessions would help in resolving certain issues, such as confusion about the stakeholders’ roles (especially local government officials and community members) in DRR, thus ensuring both buy-in on the part of all the stakeholders and also that stakeholder input is prioritised and encouraged.
- The Municipal Disaster Management Centre should enforce the implementation of the existing legal instruments that enable public participation in municipal departments to ensure that the involvement of other stakeholders, such as community members, in its DRR initiatives is prioritised to ensure that its activities are practically and effectively implemented.
- The link between members of the Municipal Disaster Management Advisory Forum and their colleagues in the various municipal departments should be strengthened. This is necessary because it would appear that some of the respondents at the municipal department were not well informed about disaster risk management despite the fact that their functions were aimed at reducing disaster risks.
- The Municipal Disaster Management Centre and other stakeholders should find ways in which to encourage community members to participate in DRR initiatives and activities and empower them by recognising their input into the decision-making process. This can be done by having an official present when Ward Councillors are holding community meetings.
- Awareness campaigns conducted by the Municipal Disaster Management Centre and other stakeholders should not be directed at schools and customer care centres in Katlehong Township only but should also conduct door to door campaigns as some respondents did not know that awareness campaigns had not been conducted in their area. Brochures and pamphlets used should be made available in local spoken languages in addition to the English language.
- The input of community members should be prioritised and encouraged by the EMM to enable change in policy where applicable and once they know and understand their role in disaster risk management. The municipality and the community members should form a partnership in order to achieve their goals as one.

## Recommendation for further research

The study also recommends further research on the prioritisation and encouragement of public participation and DRR in municipal departments to ensure that service delivery is enhanced and improved, over a larger geographical area.

## Limitations

Limitations to this study included disaster management and other municipal department officials not participating in the study because they have tight schedules while others were of the opinion that the study does not apply to them. Another limitation included community members not willing to participate because they were not getting any incentive for participating in the study, which ultimately led to a smaller sample of data.
